# Quality of Inpatient Tuberculosis Health Care in High-Burden Resource-Limited Settings: Protocol for a Comprehensive Mixed Methods Assessment Study

**DOI:** 10.2196/13903

**Published:** 2020-01-07

**Authors:** Nune Truzyan, Zaruhi Grigoryan, Lusine Musheghyan, Byron Crape, Varduhi Petrosyan

**Affiliations:** 1 Avedisian Onanian Center for Health Services Research & Development Turpanjian School of Public Health American University of Armenia Yerevan Armenia; 2 Public Health Program Scientific Reasoning Blocks Nazarbayev University School of Medicine Astana Kazakhstan

**Keywords:** tuberculosis, standard of care, health services, research design

## Abstract

**Background:**

The quality of care for tuberculosis (TB) is deficient in high-burden countries and urgently needs improvement. However, comprehensively identifying the required improvements is challenging. Providing high-quality TB care is an important step toward improving patients’ quality of life and decreasing TB morbidity and mortality. Effective tools for assessing the quality of TB services using international standards and guidelines can identify existing gaps in services and inform improvements to ensure high-quality inpatient TB services.

**Objective:**

This study aimed to develop evaluation instruments for defining the quality of provision of TB services.

**Methods:**

To assess quality of services in the largest TB hospital in Armenia, we developed instruments based on the Joint Commission International Accreditation Standards for Hospitals, International Standards for TB Care, TB Laboratories Bio-Safety Standards, and the World Health Organization framework for conducting TB program reviews. A mixed methods approach was utilized, triangulating quantitative (checklists) and qualitative (in-depth interviews) results. A scoring system and strengths, weaknesses, opportunities, and treats analysis was applied to detail results for each of the 122 standards assessed. A scaling approach was used to present overall performances of inpatient services for eight patient-centered functions and five organization management functions.

**Results:**

Overall, 40 in-depth interviews and 91 checklists (21 observations, 16 policy papers, 20 staff qualification documents, and 34 medical records) were developed, utilized, and analyzed to explore practices of health care professionals, assess inpatient treatment experience of patients and their family members, evaluate facility environmental conditions, and define the degree of compliance to standards.

**Conclusions:**

The effective comprehensive evaluation instruments and methods developed in this study for quality of inpatient TB services support the implementation of similar effective assessments in other countries. It may also become a platform to develop similar approaches for assessing ambulatory TB services in resource-limited countries.

**International Registered Report Identifier (IRRID):**

DERR1-10.2196/13903

## Introduction

### Background

In 2014, the 67th World Health Assembly set a goal for the year 2035: The number of tuberculosis (TB) deaths will reduce by 95%, the TB incidence rates will reduce by 90% as compared with those in the year 2015, and no family will “face catastrophic costs due to tuberculosis” [1]. The same report identified a stubborn persistence of the burden of TB disease in resource-limited countries, exasperated by multidrug resistant (MDR)-TB. Multidrug resistance threatens effective TB control and is a major threat to global health security. However, the effective management of MDR-TB is limited by health service barriers [1]. Approximately 39% of the estimated TB cases and 75% of the estimated MDR-TB cases globally were either undiagnosed or underreported in 2017, indicating deficiencies in the quality of TB diagnostic and treatment services [2-4].

To achieve the goal of reducing the burden of TB worldwide, the special challenges of MDR-TB in high-burden resource-limited countries such as Armenia have provided impetus for new recommendations, moving from a focus on expanding coverage of free TB diagnostic and treatment services to a focus on quality of services [5]. Provisions to achieve high-quality TB health care are essential steps toward improving TB medical practice and patient outcomes, leading to decreases in TB disease incidence [6,7]. The World Health Organization (WHO) defines high quality of health care as a patient-centered conjunction of six dimensions: effectiveness, efficiency, accessibility, acceptability, equitability, and safety. Best practices address all these dimensions [8].

### Standards Measuring Quality of Inpatient Services

The internationally adopted Joint Commission International (JCI) Accreditation Standards for Hospitals are evidence-based standards to measure the quality of services provided in hospitals in order to improve performance and outcomes of hospitals [9]. The assessment of service quality provided in hospitals is conducted through utilization of functions, standards, and measurable elements [9]. Functions consist of various standards, and the standards consist of measurable elements. Measurable elements measure the degree of compliance of hospital performance to their respective standards. Compliance to standards are, in turn, used to evaluate overall hospital performance for the respective function [9].

The Quality Improvement Handbook for TB and MDR-TB Programs identifies three perspectives on the quality of TB care: the perspectives of patients, service providers, and health facility managers [10]. Service providers’ perspective of quality [11,12] includes clinical competence, confidence, being respectful, educating patients, application of TB management core principles, team working ability, motivation, and proper documentation of treatment outcomes [13,14]. Facility managers’ perspective includes offering services that satisfy patients and community, succeeding on performing TB monitoring indicators, and assuring recognition of their health facility by other stakeholders [10]. The International Standards for TB Care instrument addresses these perspectives, which are utilized to measure the quality of care for TB patients [7].

Assessments of quality of TB health care have typically relied heavily on quantitative assessment methods, including survey instruments, checklists using simulations, checklists for direct observations in the health care facility, and chart reviews or audits [5]. However, quantitative assessments alone provide an incomplete profile of the quality of health care facility services and do not fully address underlying factors influencing quality [5,15]. Qualitative assessments such as semistructured in-depth interviews and focus groups have long been touted for answering quality of health care questions that quantitative methods are ill suited to answer [15], including questions on how health care services are actually operating [5,15]. Qualitative assessments are better at extricating reasons for questionable clinical practices and providing further clarification on how patients and caregivers experience and perceive their health care [15]. However, these qualitative assessment methods are infrequently used to assess the quality of health care [16]. They are especially absent in resource-limited high-burden countries for assessing the quality of TB health services, where there is a heavy reliance on quantitative assessment methods alone [16-20]. In addition, a large majority of the quality assurance tools for resource-limited settings are designed for national TB programs or for national- or district-level services and are not applicable to individual TB hospitals [19].

### Study Rationale

The most comprehensive understanding of the quality of health care and underlying factors influencing quality requires a combination of both quantitative and qualitative assessment methods, a *mixed methods* approach [21,22]. Mixed methods better inform the design and development of more successful interventions to improve the quality of health care [23,24]. Yet, no previous published literature integrated both qualitative and quantitative assessments to evaluate the quality of inpatient TB health care in resource-limited high-burden settings. We designed and applied a mixed methods assessment based on the WHO best practices for a comprehensive evaluation of TB inpatient health care services. This assessment was designed to inform systematic feasible improvements in quality and address the two pillars of integrated patient-centered care, and it intensified research and innovation of the Global End TB strategy [25]. This study aimed to develop evaluation instruments using international experience and different assessment tools for defining the quality of care in the largest TB inpatient facility in Armenia.

## Methods

### Study Design and Instruments

For a larger, more comprehensive, valid evaluation of the quality of diagnostic and treatment services of the largest TB hospital of the National Tuberculosis Control Center (NTCC) in Armenia, we used a mixed methods study design. We integrated qualitative and quantitative methods by triangulating results from all data collection instruments and methods [26].

**Figure 1:**
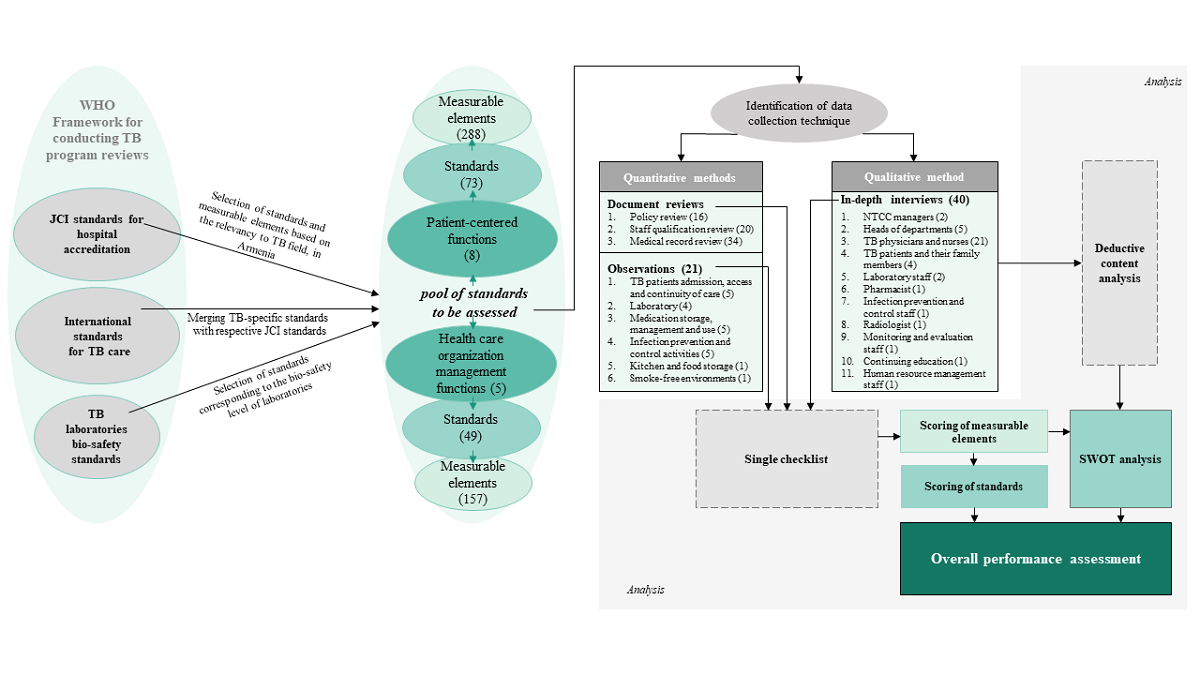
Flowchart for data management and analyses. JCI: Joint Commission International; NTCC: National Tuberculosis Control Center; SWOT: strengths, weaknesses, opportunities, and threats; TB: tuberculosis; WHO: World Health Organization.

We developed the study instruments based on suitable JCI Standards for Hospital Accreditation, International Standards for TB Care, TB Laboratories Bio-Safety Standards, and WHO Framework for Conducting TB Programs Review [9,27]. JCI standards and measurable elements were incorporated into the study instruments when appropriate for the assessment (Figure 1). These standards were divided into two main sections consisting of eight patient-centered functions, including the TB-tobacco control function [28] (using the WHO recommendations for integration of TB and tobacco control measures [29]), and five health care organization management functions. All these functions have their specific standards, and each of these standards consist of several measurable elements (Multimedia Appendix 1).

The study instruments include document review checklists for policy review, staff qualification review, and medical records; observation checklists for TB patients’ admission, access and continuity of care, laboratory services, medication storage management and use, infection prevention and control, and kitchen and food storage; and in-depth interview guides for 11 groups of key informants (Figure 1). The JCI and WHO standards or measurable elements were used to develop open-ended questions for the in-depth interview guides. All the study instruments were developed in English and translated into Armenian. The quantitative checklists were pretested before data collection; the qualitative guides were continuously improved, as needed, during the process of data collection.

The Institutional Review Board of the American University of Armenia approved the study for compliance with locally and internationally accepted ethical standards (protocol number: AUA-2016-002).

All participants were informed of their rights; all those who chose to participate provided verbal informed consent. Audio recording and observations were possible only with permission of participants; if a participant did not want to be audio recorded, only written notes were taken.

### Document Review

Policy documents were reviewed to understand the formal documents that regulate the hospital’s daily practices in relation to TB treatment and diagnosis. Reviewed documents included organizational charter of the NTCC, internal disciplinary rules of the organization, and internal regulations of different structural units. We also reviewed several guidelines and national regulations, such as hand-hygiene guidelines, waste and expired materials’ disposal guidelines, methodological guides for TB infection control, and the national standards for TB treatment and diagnosis.

The staff’s qualification document review was designed to evaluate compliance of relevant professional experience, qualifications, and credentials required for job duties and responsibilities of the staff.

Medical records review included reviews of medical records and TB treatment cards of patients with TB admitted for TB inpatient treatment at the NTCC 2 months before the assessment. We selected 2 months before the assessment to have adequate time to review the full range of medical records per patient (medical history, TB treatment card, and MDR-TB treatment files). After discharge, patients’ TB treatment cards are transferred to TB outpatient centers. All clinical departments that did not admit new TB patients during the data collection period were asked to provide all records they had from 2016.

### Observations

Observations were designed to explore several environmental conditions and daily practices of health care providers in TB treatment and diagnosis using standardized checklists. To evaluate the quality of diagnostic laboratory services, we observed daily practices in both bacteriological or microbiological and clinical laboratories of the hospital, considering the degree of compliance with the WHO biosafety standards of TB laboratories [30] and the radiology department. We used a standardized checklist to assess smoking practices and strategies in order to eliminate indoor smoking based on the observation of behaviors of health care providers and patients or family members.

### In-Depth Interviews

The study team identified key informants from clinical departments and administrative units (based on experience and expertise on inpatient care) by using purposive sampling to optimize information acquisition and convenience sampling for those willing to participate, given the optimal utilization of available resources. All stakeholders of inpatient TB services were included to ensure validity. In-depth interviews included 11 key informant groups or sampling units to protect their confidentiality and provide *data triangulation* [26]: (1) NTCC managers, (2) heads of departments, (3) TB physicians and nurses, (4) patients with TB and their family members, (5) laboratory staff, (6) pharmacists, (7) infection prevention and control staff, (8) radiologists, (9) monitoring and evaluation staff, (10) continuing education staff, and (11) human resource management staff. They were further categorized as (1) administration, (2) health care providers, and (3) patients with TB and their family members. TB health care providers were physicians and nurses with professional experience of working in the inpatient unit of the NTCC. To ensure full coverage, at least one representative from each inpatient department participated in the study. Participating TB patients (their family members) had completed their intensive phase of TB treatment in the NTCC hospital and were in the continuation phase of treatment in outpatient TB centers. For patients from the children’s TB department, only adult caregivers were contacted to participate. To recruit patients with TB, we collaborated with physicians from the TB outpatient center who made the initial contact with patients to share their contacts with the research team, and after they agreed, they passed the patients’ contact information to the research team.

### Data Management

The quantitative assessment checklists (legal and staff qualification documents review, medical records, and observations) data were entered in a Microsoft Excel 2013 worksheet for further analysis.

The qualitative in-depth interview data were analyzed utilizing deductive content analysis with a structured matrix [31-33]. The research team used a predefined structure of initial coding, which comprised the measurable elements of the selected standards (Figure 1). After verbatim transcription of the data, two researchers reviewed all transcripts and started the analysis using *investigator triangulation*. *Data triangulation* was applied across the different data sources [26].

To integrate qualitative and quantitative results to measure compliance to the standards, we developed a single checklist comprising all assessed standards with their measurable elements. Using this checklist, we applied integrated *methodological triangulation* across both quantitative and qualitative results [26].

Next, we developed a scoring system, setting the maximum score for each standard to 10. Applying weighted scores to measurable elements within each standard, we calculated the score of the assessed standards by summing the scores of their measurable elements. The number of measurable elements for each standard ranged from 2 to 10 (average=4), depending on the standards’ complexity. We based our evaluation of compliance to the NTCC’s daily practices on the obtained scores (scored from 0 to 10) for each assessed standard.

After developing the coding scheme and the scoring system, we conducted a strengths, weaknesses, opportunities, and threats (SWOT) analysis, grouping all the findings into SWOT for each of the 122 standards [34]. The findings include both a scoring table and a SWOT analysis for each of the standards. The scoring table and SWOT analysis were supported by direct quotes from respondents, which reduced the influence of biases of the study team and enhanced the findings for improved communication to a wider audience (Multimedia Appendix 2).

Furthermore, to measure the overall performance of inpatient services of the NTCC in meeting the 13 assessed functions, we calculated the *function mean score*. We calculated this score based on a scoring system we developed that identifies the level to which standards of each function were met. The score ranges were defined using the SD calculated from the mean [33], with the minimum score equal to 0 and the maximum score equal to 10. These numeric values were further defined as a function scale, with categories of *not met* (0), *minimally met* (0.1-3.3), *partially met* (3.4-6.6), *satisfactory met* (6.7-9.9), and *fully met* (10). Regarding data on meeting the standards, we have converted the scores to corresponding percentage (Multimedia Appendix 2).

## Results

### Document Review

Overall, 16 different internal policy papers and national regulations were reviewed to complete the policy review checklist. We used the policy review checklist to assess the presence or absence of certain policies and procedures that are recommended internationally. The staff’s qualification documents review utilized 20 personnel files, including descriptions of positions of all employees from all departments and the staffing plan of the organization for which standardized checklists were utilized. The medical records review included 34 medical records and TB treatment cards of patients with TB and utilized standardized checklists (Figure 1).

### Observations

Applying the standardized checklists, we conducted 21 observations in the hospital and in its vicinity (Figure 1).

### In-Depth Interviews

Overall, 40 in-depth interviews of key informants were conducted: NTCC managers (n=2), heads of departments (n=5), TB physicians and nurses (n=21), TB patients and their family members (n=4), laboratory staff (n=2), pharmacist (n=1), infection prevention and control staff (n=1), radiologist (n=1), monitoring and evaluation staff (n=1), continuing education staff (n=1), and human resource management staff (n=1; Figure 1).

### Data Management

The calculated scores of the 122 assessed standards and their SWOT analysis contributed to understanding the details and the extent to which inpatient TB services in Armenia comply with local and international standards. The scaling approach, which was applied to evaluate the overall performance of inpatient services for its 13 functions, helped visualize existing gaps in patient-centered and organization management levels of the system and share findings with a wider audience.

## Discussion

### Principal Findings

Improving the quality of TB health services is possible through adherence to international standards adapted for the local resource-limited context. Modern mechanisms of patient safety and quality assurance in inpatient and diagnostic facilities will result in sustained improvements in operations and improved quality of care provision and will create a safer environment for patients with TB including those with drug-resistant TB. The suggested protocol for quality assessment could help identify gaps in quality of care and patient safety; addressing those gaps could strengthen *the response of health systems in providing accessible, affordable, and acceptable services with patient-centered approaches* in line with the *WHO Roadmap to prevent and combat drug-resistant TB in the European region* [35].

### Conclusions

National TB programs in other countries (beside Armenia) can use similar innovative mixed methods and instruments to determine compliance of their TB care systems with the internal policies and procedures and national and international guidelines to improve TB care.

Moreover, this approach of inpatient assessment of TB services can be applied for developing and adopting mechanisms for ambulatory assessment of TB services, providing resource-limited national TB programs with a tool to comprehensively measure compliance of TB services with the international standards.

## Appendix

Multimedia Appendix 1 Standards of patient-centered and health care organization management functions to evaluate the quality of inpatient diagnostic and treatment services.

Multimedia Appendix 2 Examples of strengths, weaknesses, opportunities, and threats and overall performance analyses.

## Abbreviations

JCI: Joint Commission International
MDR: multidrug resistant
NTCC: National Tuberculosis Control Center
SWOT: strengths, weaknesses, opportunities, and threats
TB: tuberculosis
WHO: World Health Organization
