# Supplementary material for: Quality of Inpatient Tuberculosis Health Care in High-Burden Resource-Limited Settings: Protocol for a Comprehensive Mixed Methods Assessment Study
Source: JMIR Res Protoc. 2020 Jan 7;9(1):e13903. doi: 10.2196/13903 (PMC6996743; doi:10.2196/13903)
Supplement: Multimedia Appendix 1 [file resprot_v9i1e13903_app1.docx]

Multimedia Appendix 1. Standards of patient-centered and health care organization management functions to evaluate the quality of in-patient diagnostic and treatment services.


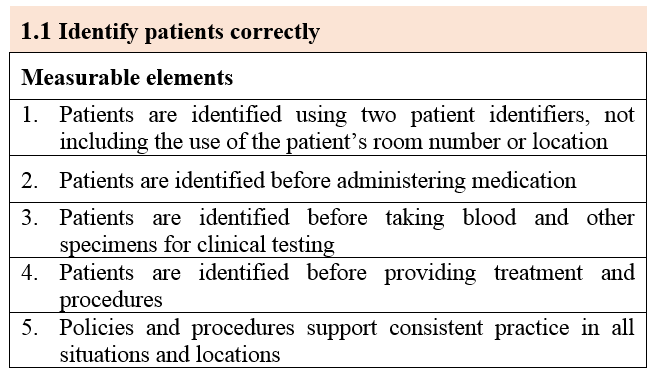


| **Patient-centered functions** |
| --- |
| 1. **International patient safety goals (IPSG)** |
| - 1. Identify patients correctly. |
| - 1. Improve effective communication. |
| - 1. Reduce risk of health care-associated infections. |
|  |
| 1. **Access and continuity of care (ACC)** |
| - 1. Patients are admitted to receive in-patient care or registered for outpatient services based on their identified health care needs and the organization’s mission and resources. |
| - 1. The organization has a process for admitting in-patients and for registering outpatients. |
| - 1. At admission as an in-patient, patients and families receive information on the proposed care, the expected outcomes of care, and any expected cost to the patient of care. |
| - 1. The organization designs and carries out processes to provide continuity of patient care services in the organization and coordination among health care practitioners. |
| - 1. There is a policy guiding the discharge of patients. |
| - 1. The clinical records of in-patients contain a copy of the discharge summary. |
| - 1. The discharge summary of in-patients is complete. |
| - 1. The receiving organization is given a written summary of the patient’s clinical condition and the interventions provided by the referring organization. |
|  |
| 1. **Patients and family rights (PFR)** |
| - 1. The organization is responsible for providing processes that support patients’ and families’ rights during care. |
| - 1. Care is respectful of the patient’s need for privacy. |
| - 1. Children, disabled individuals, the elderly, and other populations at risk receive appropriate protection. |
| - 1. Patient information is confidential. |
| - 1. The organization supports the patient’s right to respectful and compassionate care at the end of life. |
| - 1. All patients are informed about their rights and responsibilities in a manner and language they can understand. |
| - 1. Patient informed consent is obtained through a process defined by the organization and carried out by trained staff in a language the patient can understand. |
| - 1. The organization establishes a process, within the context of existing law and culture, for when others can grant consent. |
| - 1. Informed consent is obtained before surgery, anesthesia, use of blood and blood products, and other high-risk treatments and procedures. |
|  |
| 1. **Assessment of patients (AOP)** |
| - 1. All patients cared for by the organization have their health care needs identified through an established assessment process. |
| - 1. The organization has determined the minimum content of assessment, based on applicable laws and regulations and professional standards. |
| - 1. Each patient’s initial assessment(s) includes an evaluation of physical, psychological, social, and economic factors, including a physical examination and health history. |
| - 1. The patient’s medical needs are identified assessments and recorded in the clinical record. |
| - 1. Assessments are completed in the time frame prescribed by the organization. |
| - 1. Assessment findings are documented in the patient’s record and readily available to those responsible for the patient’s care. |
| - 1. All patients are reassessed at intervals based on their condition and treatment to determine their response to treatment and to plan for continued treatment or discharge. |
| - 1. Medical, nursing and other individuals and services responsible for patient care collaborate to analyze and to integrate patient assessments. |
| - 1. Laboratory services are available to meet patient needs, and all such services meet applicable local and national standards, laws, and regulations. |
| - 1. A laboratory safety program is in place, followed and documented. |
| - 1. Individuals with proper qualifications and experience administer the test and interpret the results. Laboratory results are available in a timely way as defined by the organization. |
| - 1. All equipment used for laboratory testing is regularly inspected, maintained and calibrated and appropriate records are maintained for these activities. |
| - 1. Essential reagents and other supplies are regularly available and evaluated to ensure accuracy and precision of results. |
| - 1. Established norms and ranges are used to interpret and to report clinical laboratory results. |
| - 1. A qualified individual(s) is responsible for managing the clinical laboratory service or pathology service. |
| - 1. Quality control procedures are in place, followed, and documented. |
| - 1. Laboratory access. |
| - 1. Personal protective equipment. |
| - 1. Procedures. |
| - 1. Work areas and design. |
| - 1. A radiation safety program is in place, followed and documented. |
| - 1. Individuals with proper qualifications and experience perform diagnostic imaging studies, interpret the results and report the results. Radiology and diagnostic imaging study results are available in a timely way as defined by the organization. |
| - 1. All equipment used to conduct radiology and diagnostic imaging study is regularly inspected, maintained and calibrated and appropriate records are maintained for these activities. |
| - 1. A qualified individual(s) is responsible for managing the radiology and diagnostic imaging services. |
| - 1. Quality control procedures are in place, followed, and documented. |
|  |
| 1. **Care of patients (COP)** |
| - 1. Policies and procedures and applicable laws and regulations guide the uniform care of all patients. |
| - 1. There is a process to integrate the care provided to each patient. |
| - 1. The care provided to each patient is planned and written in the patients’ record. |
| - 1. Those permitted to write patient orders write the order in the patient record in a uniform location. |
| - 1. Policies and procedures guide the care of high-risk patients and the provision of high-risk services. |
| - 1. A variety of food choices, appropriate for the patient’s nutritional status and consistent with his or her clinical care, is regularly available. Food preparation, handling, storage, and distribution are safe and comply with laws, regulations, and current acceptable practices. |
|  |
| 1. **Medication management and use (MMU)** |
| - 1. Medication use in the organization complies with applicable laws and regulations and is organized to meet patient needs. |
| - 1. Supervision of the pharmacy or pharmaceutical service. There is a method for overseeing the organization’s medication list and medication use. |
| - 1. The organization can readily obtain medications not stocked or normally available to the organization or for times when the pharmacy is closed. |
| - 1. Medications are properly and safely stored. |
| - 1. Emergency medications are available, monitored and safe when stored out of pharmacy. |
| - 1. The organization has a medication recall system. |
| - 1. Prescribing, ordering, and transcribing are guided by policies and procedures. |
| - 1. Medications prescribed and administered are written in the patient’s record. |
| - 1. A system is used to dispense medications in the right dose to the right patient at the right time and in a safe and clean environment. |
| - 1. The organization identifies those qualified individuals permitted to administer medications. |
| - 1. Medication administration includes a process to verify the medication is correct based on the medication order. |
| - 1. Medication effects on patients are monitored. |
| - 1. Medication errors, including near missed, are reported through a process and time frame defined by the organization. |
|  |
| 1. **Patient and family education (PFE)** |
| - 1. The organization provides education that supports patient and family participation in care decision and care processes. |
| - 1. Each patient’s educational needs are assessed and recorded in his or her record. |
| - 1. The patient’s and family’s ability to learn and willingness to learn are assessed. |
| - 1. Patient and family education includes the following topics, related to the patient’s care: the safe use of medications, the safe use of medical equipment, potential interactions between medications and food, nutritional guidance, pain management, and rehabilitation techniques. |
| - 1. Education methods include the patient’s and family’s values and preference and allow sufficient interaction among the patient, family, and staff for learning to occur. |
| - 1. Health professionals caring for the patient collaborate to provide education. |
|  |
| 1. **TB-tobacco control (TBTC)** |
| - 1. Availability of “No smoking” signs. |
| - 1. Availability of smoking related functional items and policies in the areas where smoking is prohibited. |
| - 1. Presence of proofs related to tobacco-free environment. |
|  |
| **Health care organization management functions** |
| 1. **Quality improvement and patient safety (QPS)** |
| - 1. Those responsible for governing and managing the organization participate in planning and measuring a quality improvement and patient safety program |
| - 1. Quality improvement and patient safety information is communicated to staff. |
| - 1. The organization designs new and modified systems and processes according to quality improvement principles. |
| - 1. Clinical practice guidelines, clinical pathways, and/or clinical protocols are used to guide clinical care. |
| - 1. The organization’s leaders identify key measures in the organization’s structures, processes, and outcomes to be used in the organization wide quality improvement and patient safety plan. |
| - 1. Individuals with appropriate experience, knowledge, and skills systematically aggregate and analyze data in the organization. |
| - 1. The analysis process includes comparisons internally, with other organizations when available, and with scientific standards and desirable practices. |
| - 1. The organization uses a defined process for identifying and managing sentinel events. |
|  |
| 1. **Prevention and control of infection (PCI)** |
| - 1. One or more individuals oversee all infection prevention and control activities. This individual(s) is qualified in infection prevention and control practices through education, training, experience, or certification. |
| - 1. There is a designated coordination mechanism for all infection prevention and control activities that involves physicians, nurses, and others based on the size and complexity of the organization. |
| - 1. The infection prevention and control program is based on current scientific knowledge, accepted practice guidelines, applicable laws and regulations, and standards for sanitation and cleanliness. |
| - 1. The organization’s leaders provide adequate resources to support the infection prevention and control program. |
| - 1. The organization designs and implements a comprehensive program to reduce the risks of health care–associated infections in patients and health care workers. |
| - 1. All patient, staff, and visitor areas of the organization are included in the infection prevention and control program. |
| - 1. The organization identifies the procedures and processes associated with the risk of infection and implements strategies to reduce infection risk. |
| - 1. There is a policy and procedure in place that identifies the process for managing expired supplies and defines the conditions for reuse of single-use devices when laws and regulations permit. |
| - 1. The organization has a policy and procedure on the disposal of sharps and needles. |
| - 1. Gloves, masks, eye protection, other protective equipment, soap, and disinfectants are available and used correctly when required. |
| - 1. The organization tracks infection risks, infection rates, and trends in health care–associated infections. |
| - 1. The results of infection prevention and control measurement in the organization are regularly communicated to leaders and staff. |
| - 1. The organization provides education on infection prevention and control practices to staff, physicians, patients, families, and other caregivers when indicated by their involvement in care. |
|  |
| 1. **Governance, leadership, and direction (GLD)** |
| - 1. Governance responsibilities and accountabilities are described in bylaws, policies and procedures, or similar documents that guide how they are to be carried out. |
| - 1. Those responsible for governance approve and make public the organization’s mission statement. |
| - 1. Those responsible for governance approve the policies and plans to operate the organization. |
| - 1. Those responsible for governance approve the budget and allocate the resources required to meet the organization’s mission. |
| - 1. Those responsible for governance appoint the organization’s senior manager(s) or director(s). |
| - 1. A senior manager or director is responsible for operating the organization and complying with applicable laws and regulations. |
| - 1. Organization leaders ensure that there are uniform programs for the recruitment, retention, development, and continuing education of all staff. |
| - 1. Medical, nursing, and other leaders of clinical services plan and implement an effective organizational structure to support their responsibilities and authority. |
| - 1. Directors recommend space, equipment, staffing, and other resources needed by the department or service. |
| - 1. Directors recommend criteria for selecting the department or service’s professional staff and choose or recommend individuals who meet those criteria. |
| - 1. Directors provide orientation and training for all staff of the duties and responsibilities for the department or service to which they are assigned. |
|  |
| 1. **Staff qualifications and education (SQE)** |
| - 1. The organization uses a defined process to ensure that clinical staff knowledge and skills are consistent with patient needs and the non-clinical staff’s knowledge and skills are consistent with the organization’s needs. |
| - 1. Organization leaders define the desired education, skills, knowledge, and other requirements of all staff members. There is documented personnel information for each staff member. |
| - 1. Each staff member receives ongoing in-service and other education and training to maintain or to advance his or her skills and knowledge. |
| - 1. The organization provides a staff health and safety program. |
| - 1. The organization has an effective process for gathering, verifying, and evaluating the credentials (licensure, education, training, competence, and experience) of the medical, nursing, and other staff. |
| - 1. Leadership makes an informed decision about renewing permission for each medical staff member to continue providing patient care services at least every three years. |
|  |
| 1. **Management of communication and information (MCI)** |
| - 1. The organization informs patients and families about its care and services and how to access those services. |
| - 1. Communication is effective throughout the organization. |
| - 1. The leaders ensure that there is effective communication and coordination among those individuals and departments responsible for providing clinical services. |
| - 1. Information about the patient’s care and response to care is communicated among medical, nursing, and other health care practitioners during each staffing shift and between shifts. |
| - 1. The patient’s record(s) is available to the health care practitioners to facilitate the communication of essential information. |
| - 1. Information related to the patient’s care is transferred with the patient. |
| - 1. Information privacy and confidentiality are maintained. |
| - 1. Records and information are protected from loss, destruction, tampering, and unauthorized access or use. |
| - 1. The organization initiates and maintains a clinical record for every patient assessed or treated. |
| - 1. The clinical record contains sufficient information to identify the patient, to support the diagnosis, to justify the treatment, to document the course and results of treatment, and to promote continuity of care among health care practitioners. |
| - 1. Every patient clinical record entry identifies its author and when the entry was made in the record. |
