# Supplementary material for: Quality of Inpatient Tuberculosis Health Care in High-Burden Resource-Limited Settings: Protocol for a Comprehensive Mixed Methods Assessment Study
Source: JMIR Res Protoc. 2020 Jan 7;9(1):e13903. doi: 10.2196/13903 (PMC6996743; doi:10.2196/13903)
Supplement: Multimedia Appendix 2 [file resprot_v9i1e13903_app2.docx]

Multimedia Appendix 2. Examples of strengths, weaknesses, opportunities, and threats and overall performance analyses.

An example of a strengths, weaknesses, opportunities, and threats (SWOT) analysis for one of the standards (3.1) in the Patients and family rights (PFR) function and its performance in the inpatient unit of the National TB Control Center (NTCC) Armenia.

## Patients and family rights (PFR) function

| 1. **Standard: The organization is responsible for providing processes that support patient and family rights during care** | | |
| --- | --- | --- |
| From hospital leaders to clinical staff members should have a clear understanding of patient and family rights. Leaders should recognize patients and family rights and acknowledge their own role in protecting those rights. The leadership of the inpatient unit should convey this vision to all staff members and have all staff members educated on their responsibilities to work together to protect patient and family rights. On the other hand, family members and patients should be notified of their rights to participate in care related decisions to further protect their rights. And finally, there should be policies and procedures that cover all aspects, roles and responsibilities involved in the process of protecting patients and family rights. | | |
| **Measurable elements** | Max. score | Obtained score |
| 1. The organization’s leaders work collaboratively to protect and to advance patient and family rights. | **2.0** | **0** |
| 1. The leaders understand patient and family rights as identified in laws and regulations and in relation to the cultural practices of the community or individual patients served. | **2.0** | **1.0** |
| 1. The organization respects the right of patients, and in some circumstances the right of the patient’s family, to have the prerogative to determine what information regarding their care would be provided to family or others, and under what circumstances. | **2.0** | **2.0** |
| 1. Staff members are knowledgeable about the policies and procedures related to patient rights and can explain their responsibilities in protecting patient rights. | **2.0** | **0** |
| 1. Policies and procedures guide and support patient and family rights in the organization. | **2.0** | **0** |
| Total score | **10.0** | **3.0** |

**Strengths.** During the in-depth interviews (IDIs), nearly all clinical staff members highlighted their respect towards patient and family rights and their prerogative to choose what health care related information is to be provided to family members or others and under what circumstances, despite the absence of formally introduced procedures. In the DR-TB department, which has collaborated for years with the MSF France, all health care providers received oral instructions from the department staff members on PFR; moreover, the departmental staff signed contracts with patients and obtained their written consent before initiating the treatment.

| *A social assessment form is used to assess the drug-resistant patients. After that they sign a contract, where all their rights and responsibilities are described. We have a social worker, who is responsible for informing patients on that.*  **Health care provider**  *Patients know their rights very well and we try to do our best to have them feel good...*  **Health care provider** |
| --- |

**Weaknesses.** The hospital leadership put insufficient emphasis on the importance of patient and family rights. As a result, the organization had no hospital-wide policies and procedures, enforcing practices directed to protect patient and family rights. Even the practice established by MSF France in the DR-TB department was not considered by the inpatient unit leaders as an example for developing actions toward promotion of patient and family rights throughout the hospital. Except for the DR-TB department, promotion of patient and family rights was done based on clinicians’ personal understanding of the issue at the level of oral communication or was not practiced at all.

| *We do not sign a contract with drug-susceptible TB patients, as we do for drug-resistant patients. We assure implementation of patients’ rights through our daily work with them.*  **Health care provider**  *Previously, we* [drug-susceptible TB department] *used to have a consent form. Now patients not required to sing a contract for treatment.*  **Health care provider** |
| --- |

**Opportunities.** Development of policies and procedures describing patient and family rights promotion in the inpatient unit could serve as a basis for creating an environment respecting patients and family rights. In the frames of newly developed policies and procedures the hospital leadership and the clinical staff should be educated on the principles of patient and family rights protection and promotion. This in its turn will lead to having all staff members acknowledging their role in the promotion of patients and family rights and implementation of processes described in policy or procedure documents.

**Threats.** Adding new responsibilities to the existing ones might face initial resistance from the hospital staff. However, if the hospital leadership achieves and maintains a common understanding of patient and family rights and demonstrates commitment to protecting PFR, this hurdle could be overcome.

An example of the Overall Performance analysis for PFR Function in the inpatient unit of the NTCC Armenia.

| 1. **PATIENTS AND FAMILY RIGHTS (PFR) FUNCTION** | | | | | |
| --- | --- | --- | --- | --- | --- |
| **Standards and scores met** | **Not**  (0) | **Minimally**  (0.1 - 3.3) | **Partially** (3.4 – 6.6) | **Satisfactory**  (6.7 - 9.9) | **Fully**  (10) |
| 3.1 The organization is responsible for providing processes that support patient and family rights during care |  | 3.0 |  |  |  |
| 3.2 Care is respectful of the patient’s need for privacy. |  |  |  | 7.0 |  |
| 3.3 Children, disabled individuals, the elderly, and other populations at risk receive appropriate protection |  |  | 4.5 |  |  |
| 3.4 Patient information is confidential |  |  | 6.5 |  |  |
| 3.5 The organization supports the patient’s right to respectful and compassionate care at the end of life |  |  | 5.0 |  |  |
| 3.6 All patients are informed about their rights and responsibilities in a manner and language they can understand |  |  | 6.5 |  |  |
| 3.7 Patient informed consent is obtained through a process defined by the organization and carried out by trained staff in a language the patient can understand |  | 1.0 |  |  |  |
| 3.8 The organization establishes a process, within the context of existing law and culture, for when others can grant consent |  | 2.0 |  |  |  |
| 3.9 Informed consent is obtained before surgery, anesthesia, use of blood and blood products, and other high-risk treatments and procedures | 0 |  |  |  |  |
| **Function mean score** | **3.9** | | | | |

- The National law for TB diagnosis and treatment in Armenia protected TB patient and family rights for receiving free care regardless of TB type, severity and treatment duration. The hospital staff and leaders worked to protect and advance the patient rights, primarily by maintaining confidentiality of the treatment. Physicians verbally informed patients and their families about their rights. Only MDR TB department had established written policies and procedures to guide and support patient rights by providing a written informed patient consent form and signing an agreement with the patient to undergo treatment. Policies and procedures for other departments were verbally established. A written document specifically describing PFR does not exist at the inpatient unit.
- The privacy of care was given high priority at the inpatient unit. Staff members were respectful and acted accordingly to maintain patient health information confidential.
- The National law listed the high-risk patient groups that might need provision of special services. These vulnerable groups include children, disabled individuals, and older adults and other populations at risk; for example, disabled individuals or the patients who cannot walk and only stay at home are guaranteed with home treatment, covered by the state budget. However, the hospital staff members failed to clearly understand their responsibilities to protect the vulnerable groups.
- The inpatient unit did not have a formal protocol on respectful and compassionate end-of-life care for dying patients.
- The inpatient unit performed successfully in informing patients about their diagnosis and proposed treatments. However, systematically informing patients and family of their rights and responsibilities was not in place; for example, patient consent for an inpatient treatment and/or other procedures were not obtained.
- The inpatient unit did not have written formal policies and procedures on the promotion and protection of patient and family rights.

The inpatient unit achieved 39% performance (final score converted to a percentage) for the PFR function by meeting specific standards satisfactory, partially, minimally, or not meeting at all. The overall function was graded as **partially performed** and the inpatient unit of the NTCC Armenia would require several improvements.
